# A Rare Case of Tumor-to-Tumor Metastasis: Renal Cell Carcinoma Metastasis to Papillary Thyroid Carcinoma

**DOI:** 10.1210/jcemcr/luae081

**Published:** 2024-05-21

**Authors:** Shourya Tadisina, Farhan Sami, Daniel Mettman, Maricel Ridella

**Affiliations:** Division of Endocrinology Diabetes and Metabolism, University of Missouri-Kansas City, Kansas City, MO 64108, USA; Division of Clinical Pathology, The University of Kansas, Kansas City, KS 66160, USA; Department of Clinical Pathology, Division of Endocrinology Diabetes and Metabolism, Kansas City Veterans Association Medical Center, Kansas City, MO 64128, USA; Department of Clinical Pathology, Division of Endocrinology Diabetes and Metabolism, Kansas City Veterans Association Medical Center, Kansas City, MO 64128, USA

**Keywords:** tumor-to-tumor metastasis, papillary thyroid carcinoma, renal cell carcinoma

## Abstract

Papillary thyroid carcinoma (PTC) is the most common thyroid malignancy. Renal cell carcinoma (RCC) metastasis to the thyroid, albeit the most common carcinomatous metastasis to the thyroid, is rare, and tumor-to-tumor metastasis of RCC to PTC is even rarer. We present a case of a 65-year-old male with a history of RCC who presented with a thyroid nodule 7 years after left radical nephrectomy. Imaging showed the thyroid nodule predating the kidney tumor. Fine-needle aspiration biopsy was performed and showed 2 distinct cell populations, 1 of which was stained with RCC markers and another that was stained positively for thyroid markers. An interpretation of atypia of undetermined significance was rendered and molecular testing was indeterminate with ThyGeNEXT not detecting mutations and ThyraMIR positive for a level 2 microRNA pattern consistent with low risk for malignancy. The patient elected for active surveillance until follow-up thyroid ultrasound showed continued growth. At this point, a right hemithyroidectomy was performed. Pathology confirmed clear cell RCC metastasis to an infiltrative follicular variant papillary thyroid carcinoma. This case highlights the possibility of tumor-to-tumor metastasis in patients with a previous history of RCC even years after nephrectomy and in the absence of other metastatic lesions.

## Introduction

Metastasis to the thyroid gland by extrathyroidal malignancies is uncommon and accounts for only 2% to 3% of thyroid malignancies (1-3). Renal cell carcinoma (RCC) is the most frequent malignancy to metastasize to the thyroid. RCC is notorious for metastasizing to unusual sites and recurring years after nephrectomy in about one-third of cases (4). In comparison to the frequency of neoplasia, tumor-to-tumor metastasis is extremely rare (5). RCC is also the most frequent malignancy to metastasize to thyroid neoplasms (5). Herein, we report a case of an RCC metastasis to a papillary thyroid carcinoma and provide a literature review of the 11 previously reported cases.

## Case Presentation

A 65-year-old male with a history of RCC, type 2 diabetes mellitus, and thyroid nodules was referred to our endocrinology service by his primary care physician. RCC had been diagnosed 7 years prior and was treated with a left radical nephrectomy. The patient's clinicopathological staging was pT3aNx (AJCC-UICC8). Per American Urological Association guidelines, he was followed closely with serial imaging, which up to this point had not shown signs of recurrence. He had been told nearly a decade prior that he had thyroid nodules, but neither follow-up imaging nor a biopsy was ever performed. He denied any hypothyroid, hyperthyroid, or compressive symptoms. Vital signs were normal. Physical exam revealed thyromegaly without a palpably distinct nodules, and the remainder of the exam was unremarkable.

## Diagnostic Assessment

**Figure 1.**
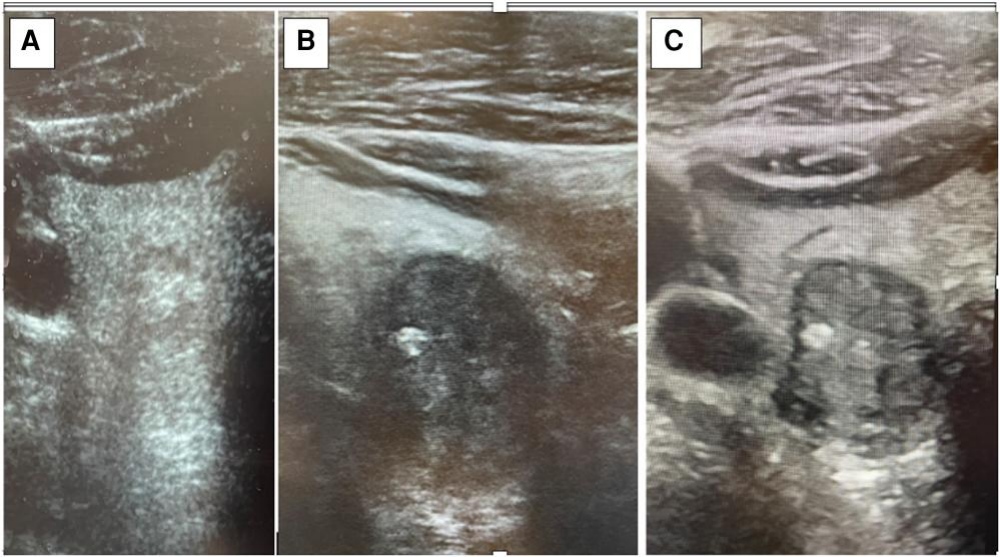
US images showing progression of the right inferior thyroid nodule. Initial US from 2006 (A), 10 years prior to nephrectomy, shows nodule to be 0.9 × 0.9 × 1.0 cm. Follow-up US in 2021 (B) shows the nodule to be solid, hypoechoic, taller than wide, with ill-defined margins in some areas with likely macrocalcifications and measuring 2.1 × 1.8 × 1.8 cm. Follow-up US in 2022 (C) shows the nodule to increase in size and measure 2.3 × 2 × 1.8 cm, which led to electing for the right hemithyroidectomy. Abbreviations: US, ultrasound.

**Figure 2.**
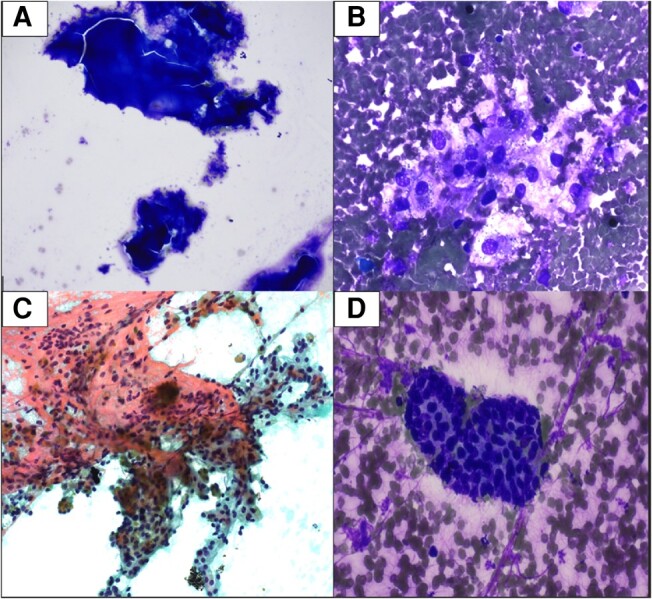
The fine-needle aspiration biopsy smears. (A) Benign follicular nodule; (B) clear cells on diff-quik stain 400× (40× objective,10× ocular); (C) clear cells on papanicolaou stain 400× (40× objective, 10× ocular); (D) atypical follicular cells on diff-quik stain 400× (40× objective, 10× ocular).

Thyroid ultrasound (US) revealed subcentimeter nodules on the left thyroid and a 2.1 cm TIRADS 5 nodule on the right inferior thyroid (Fig. 1). The right nodule was biopsied by fine needle aspiration (FNA). Smears showed abundant colloid and a few follicular cells (Fig. 2A), so a diagnosis of benign follicular nodule was rendered. Due to high clinical suspicion for malignancy, a repeat FNA was performed 2 months later. The smear and cell block revealed 2 populations of cells, atypical epithelial cells with clear cytoplasm (Fig. 2B and 2C), and another of mild atypical follicular cells (Fig. 2D). The follicular cells were mildly atypical in that they occurred in tight clusters. The clear cells were atypical in that they had large, irregular nuclei with abundant clear cytoplasm. Immunostains on the cell block showed the clear cells to be positive for carbonic anhydrase IX (CAIX) and the follicular cells to be positive for thyroid transcription factor-1 (TTF-1). In light of the patient's history of RCC, this pattern of staining prompted consideration of metastatic RCC to the thyroid. However, clear cell morphology and CAIX staining are also seen in a variety of benign and malignant thyroid lesions (6, 7). The paucicellularity of our specimen in conjunction with the imperfect specificity of the morphology and immunophenotype prompted an interpretation of atypia of undetermined significance (Bethesda III). Molecular testing was done with ThyGeNEXT not detecting any mutations and ThyraMIR positive (level 2 microRNA pattern). Together, these findings conferred a 15% to 20% risk of malignancy. The options of surgical lobectomy vs surveillance imaging were discussed with the patient, and he opted for surveillance imaging. Six months later a follow-up thyroid US showed a 20% increase in size of the nodule in 2 dimensions with a max diameter of 2.3 cm (Fig. 1); the US was negative for suspicious lymph nodes. A right hemithyroidectomy was performed, and gross examination revealed a 3-cm mass.

**Figure 3.**
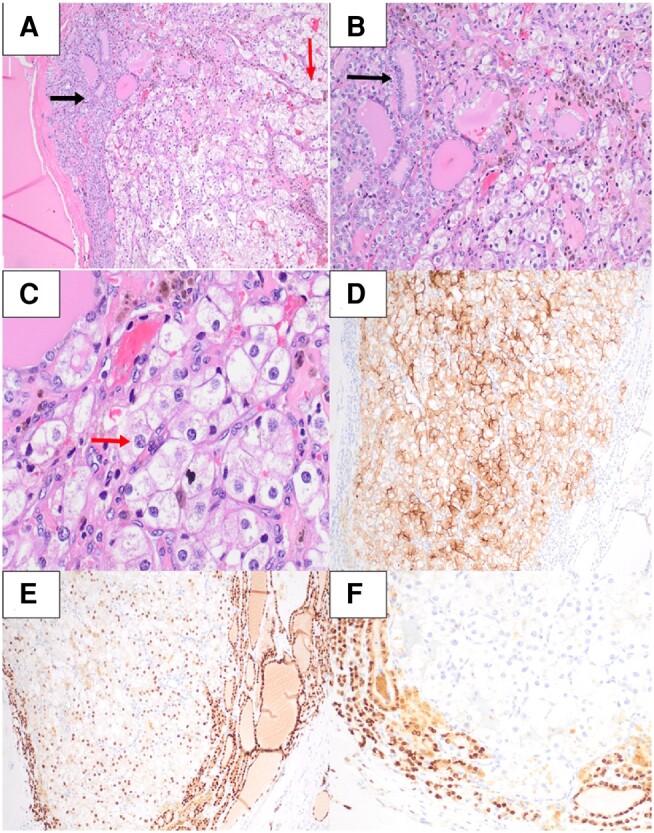
Thyroidectomy histologic images. (A) Neoplastic follicular cells (black arrow) surrounding a population of clear cells (red arrow), H&E-stained section at 100× (10× objective, 10× ocular). (B) Neoplastic follicular cells exhibit follicular variant papillary nuclear features (ie, nuclear membrane irregularities, overlapping, and clearing) (black arrow) (H&E; 200X). (C) Clear cells with abundant clear cytoplasm and nuclei containing prominent nucleoli (red arrow) rather than papillary nuclear features (H&E; 400X). (D) Both cell types were positive for PAX-8 by IHC (100X). (E) The clear cells showed membranous CD10 positivity by IHC (100X). (F) The follicular cells showed TTF-1 positivity by IHC (200×). Abbreviations: H&E, hematoxylin and eosin; IHC, immunohistochemistry; TTF-1, thyroid transcription factor.

Histologic examination of the mass revealed it to be circumscribed by a rim of follicles with papillary nuclei (ie, enlargement, overlapping, nuclear membrane irregularities, powdery chromatin, and “Orphan Annie” clearing) of the follicular type (ie, less elongation and fewer intranuclear cytoplasmic pseudoinclusions) (Fig. 3). Immunohistochemistry (IHC) showed the peripheral cells to stain positively for paired box gene 8 (PAX-8) and TTF-1 and negatively for CD10 (Fig. 3). The mass was unencapsulated with extensions of papillary carcinoma into adjacent tissue. Altogether, the morphology and immunoprofile are compatible with infiltrative follicular variant of papillary thyroid carcinoma (IFVPTC). The papillary thyroid carcinoma (PTC) component of the primary tumor was 3.0 cm. There was no evidence of angioivasion, lymphatic invasion, or extrathyroidal extension. The resection margins were free of tumor. Meanwhile, the center of the mass was predominantly composed of nests of cells with abundant clear cytoplasm (Fig. 3). IHC showed the central cells to stain positively for PAX-8 and CD10 and negatively for TTF-1 (Fig. 3). The patient history, tumor morphology, and immunoprofile were suggestive of metastatic clear cell renal cell carcinoma (ccRCC). Subsequent next-generation sequencing with the Tempus xT assay effectively confirmed the diagnosis by detecting *VHL* and *PBRM1* variants.

## Treatment

Surgical pathology confirmed tumor-to-tumor metastasis of ccRCC and IFVPTC with a total size of 3.0 cm. Based on review of previous imaging, the right thyroid nodule was noted to have been present for over a decade, many years prior to diagnosis of RCC. At the time of initial detection, the thyroid nodule was of insufficient size to warrant biopsy, and the patient was not seen by endocrinology nor was the nodule imaged again until 7 years after left radical nephrectomy, at which point the nodule met criteria for biopsy. As the patient had bilateral thyroid nodules years before the RCC was first detected, completion thyroidectomy was performed to determine if RCC has metastasized to any of the left thyroid nodules. The left side was negative for RCC but contained multiple papillary microcarcinomas.

## Outcome and Follow-up

The patient was referred to oncology after the tumor-to-tumor metastasis was diagnosed. Staging via computed tomography of the chest, abdomen, and pelvis as well as magnetic resonance imaging of the brain was negative for other sites of metastasis. He is currently being followed closely with surveillance serial imaging. Levothyroxine replacement was started after completion of thyroidectomy, and 6-week- follow-up labs showed a thyroglobulin level of <0.1 ng/mL(<0.1 ug/L), with negative thyroglobulin antibodies, and TSH-0.181 uIU/mL (0.181 mIU/L) He is receiving levothyroxine treatment to maintain his goal TSH between 0.1 and 0.5 mIU/L with close monitoring of thyroglobulin levels.

## Discussion

In the English literature, only 11 cases of RCC metastasizing to thyroid neoplasms have been reported (Table 1). RCC is both the most frequent malignancy to metastasize to the thyroid as well as the most frequent malignancy to metastasize to thyroid neoplasms (5). FVPTC is noted to be the most common recipient thyroid neoplasm for tumor-to-tumor metastasis based on the reported cases. It is postulated that the rich blood supply of the thyroid gland and thyroid tumors increase the propensity of the thyroid gland relative to other organs to be a recipient for metastatic deposits from other tumors (5). The interval from RCC diagnosis is quite variable with 1 case being diagnosed synchronously (11) and 14 years (4) being the longest reported interval. Among the reviewed cases, most of these cases have reported solitary tumor-to-tumor metastasis with no other sites involved.

**Table 1.** Summary of reported cases of RCC metastasis to thyroid gland tumors, including the type of recipient tumor, interval from nephrectomy, other sites of metastases, and IHC profile of metastatic RCC

Cases	Age/sex	Recipient tumor	Interval from RCC diagnosis and treatment	Other sites of metastasis	IHC of metastatic RCC	Follow-up and outcome
Koo et al (8)	48Y/F	FA	5Y	None	CK, CD10, and galectin (+); TTF-1, TG, and calcitonin (−)	Immunotherapy with interferon-α and interleukin-2
Rosai (cited by Ryška and Čáp (9))	NA	FA	NA	NA	TG (−)	NA
Ryška and Čáp (9)	52Y/M	Oncocytic carcinoma	13Mo	Subcutaneous	AE1/AE3, vimentin, EMA (+); TG, CEA, calcitonin (−)	NA
Baloch and LiVolsi (5)	78Y/M	FVPTC	2Y	Liver, nontumorous thyroid, pancreas	Negative for IHC staining	Received chemotherapy, developed DIC and death
Wolf et al (10)	60Y/F	MFA	2Y	NA	NA	NA
Qian et al (11)	53Y/F	Oncocytic adenoma	RCC diagnosed synchronously	None	TG, TTF-1 (−), CD10, and vimentin (+)	Nephrectomy 2 months later
Bohn et al (3)	68Y/M	PTC	2Y	Bony metastasis to multiple vertebral bodies	RCC markers(+); TG (−)	Bone metastasis at 1Y
Yu et al (12)	61Y/M	FVPTC	3Y	None	CAM 5.2, CD10, vimentin, p53 (+); TG, TTF-1, RCC Ag and p63(−)	No further therapy and tumor free at 1Y
Medas et al (13)	62Y/F	MFA	6Y	None	CD10 (+); TG, TTF-1, galectin-3(−)	Tumor free for 3Y
Kefeli and Mete (14)	80Y/F	IFVPTC	18Y	None	PAX-8, CD10 (+); vimentin (equivocal); TG, TTF-1, CEA, calcitonin (−)	NA
Badawi and Meliti (4)	63Y/F	FVPTC	14Y	Contralateral kidney	PAX-8, CAIX, galectin-3, vimentin (+); TG, TTF-1, CK7, CK19 (−)	Thyroid tissue remnant excision multiple foci of PTC
Current case	65Y/M	IFVPTC	7Y	None	PAX-8, CD10, TTF-1 (+)	Tumor free for 6 Mo

Abbreviations: CAIX, carbonic anhydrase IX; F, female; FA, follicular; IFVPTC, infiltrative follicular variant of papillary thyroid carcinoma; IHC, immunohistochemistry; M, male; Mo, months; NA, not applicable; PAX, paired box gene 8; PTC, papillary thyroid carcinoma; RCC, renal cell carcinoma; TG, thyroglobulin; TTF-1, thyroid transcription factor; Y, years.

In relation to the frequency of neoplasia, tumor-to-tumor metastasis is extremely rare. To prevent overdiagnosis, strict criteria have been developed by Dobbing (15) in 1958 and Campbell et al (16) in 1968. They require confirming the presence of 2 distinct neoplasms and the legitimacy of metastasis of 1 neoplasm to another. Specifically, these criteria exclude amalgamation of tumors by direct contiguous spread or metastases to lymph nodes involved in hematolymphoid neoplasia (15, 16). Our case meets both criteria for tumor-to-tumor metastasis. The distinct morphologies and immunoprofiles provide evidence for the presence of 2 distinct neoplasms. The thyroid nodule predating the ccRCC as well as the circumscription of the ccRCC by the thyroid neoplasm provide evidence for the ccRCC metastasizing to the IFVPTC.

The pathogenesis of tumor-to-tumor metastasis is not well understood. The rich vascularity of the thyroid gland and its primary malignancies is likely responsible for its predisposition to be a recipient for metastatic deposits from other tumors (5). Thyroid nodules in patients with a previous history of cancer can be diagnostically challenging. Most of these thyroid nodules represent a primary thyroid process rather than a metastatic process (13). It is important to consider the possibility of metastasis, especially when 2 different cell populations are identified on biopsy (17). Our case was clinically diagnosed as multinodular goiter a few years prior to diagnosis and resection of RCC, but the thyroid nodules had not been biopsied. The growth in the right thyroid nodule prompted a biopsy that showed the presence of 2 different cell populations. The pertinent history of RCC and the presence of clear cells guided us to stain the cells appropriately and plan timely intervention.

As described earlier, RCCs have very unpredictable behaviors with tremendous variability in presentations; there are reports of metastases representing the initial presentation of the RCC and about one-third of cases presenting with metastasis years after nephrectomy (1, 4, 18, 19). In our case, the tumor-to-tumor metastasis from RCC to FVPTC was discovered 7 years after nephrectomy and was the only site of metastasis. Our patient was a candidate for resection as it was the only site of metastasis. Knowledge of tumor-to-tumor metastases is of the utmost importance as it can help with diagnosis and guide patient management.

## Learning Points

- Considering the possibility of metastasis and tumor-to-tumor metastasis is essential when evaluating thyroid nodules, especially in patients with a history of RCC or in specimens with clear cell or distinct cell populations.
- On FNA, when clear cells or 2 populations of cells are observed, IHC can aid diagnosis and management.
- RCC is the most common metastatic tumor in the thyroid gland and has an unpredictable course with recurrence reported many years after nephrectomy.
- Tumor-to-tumor metastasis is diagnosed based on strict criteria by Dobbing and Campbell et al and requires the presence of 2 distinct neoplasms and true metastasis to the recipient tumor.

## Data Availability

Data sharing is not applicable to this article as no datasets were generated or analyzed during the current study.
